# Imaging dopamine function and microglia in asymptomatic LRRK2 mutation carriers

**DOI:** 10.1007/s00415-020-09830-3

**Published:** 2020-04-21

**Authors:** Morten Gersel Stokholm, Alicia Garrido, Eduardo Tolosa, Mónica Serradell, Alex Iranzo, Karen Østergaard, Per Borghammer, Arne Møller, Peter Parbo, Kristian Stær, David J. Brooks, Maria José Martí, Nicola Pavese

**Affiliations:** 1 Department of Nuclear Medicine and PET Centre, Aarhus University Hospital, Noerrebrogade 44, bldg. 10G, 8000 Aarhus C, Denmark; 2 Movement Disorders Unit, Neurology Service, Hospital Clínic de Barcelona, Barcelona, Spain; 3 Centro de Investigación Biomédica en Red Sobre Enfermedades Neurodegenerativas (CIBERNED), Hospital Clínic, IDIBAPS, Universitat de Barcelona, Barcelona, Spain; 4 Department of Neurology, Hospital Clínic de Barcelona, Barcelona, Spain; 5 Department of Neurology, Aarhus University Hospital, Aarhus C, Denmark; 6 Division of Neuroscience, Newcastle University, Newcastle upon Tyne, England

**Keywords:** Genetics, Parkinson’s disease, Clinical neurology, PET, Movement disorders

## Abstract

Neuroinflammation (microglial activation) and subclinical nigrostriatal dysfunction have been reported in subjects at risk of Parkinsonism. Eight non-manifesting carriers (NMCs) of LRRK2 G2019S mutation had ^11^C-PK11195 and ^18^F-DOPA PET to assess microglial activation and striatal dopamine system integrity, respectively. Comparisons were made with healthy controls. Five LRRK2-NMCs had subclinical reductions of putaminal ^18^F-DOPA uptake. Three of them had significantly raised nigral ^11^C-PK11195 binding bilaterally. These findings indicate that nigrostriatal dysfunction and neuroinflammation occur in LRRK2-NMCs. Studies in larger cohorts with appropriate follow-up are needed to elucidate the significance of neuroinflammation in the premotor phase of LRRK2-PD.

Mutations in the leucine-rich repeat kinase 2 gene (LRRK2) are known to cause inherited Parkinson’s disease (PD). LRRK2-associated PD (LRRK2-PD) presents with a low penetrant autosomal-dominant inheritance pattern with a cumulative risk of developing PD ranging from 26 to 80% at the age of 80 years [1, 2]. Patients with the G2019S mutation, the most common mutation in LRRK2-PD, are clinically indistinguishable from idiopathic PD and most cases present similar pathological findings with degeneration of dopaminergic neurons in the substantia nigra and occurrence of Lewy-type α-synuclein pathology [3]. Studying non-manifesting carriers (NMCs) of the LRRK2 G2019S mutation provides an opportunity to identify pathophysiological processes occurring in the premotor phase of genetic PD. This is essential to identify biomarkers and therapeutic targets to halt disease progression.

Microglia, the major resident immune cells of the central nervous system, monitors the brain milieu in their resting physiologic state, but when activated by injury, they can express both neuroprotective and cytotoxic phenotypes. It is hypothesized that a pro-inflammatory phenotype, leading to neuronal dysfunction, may drive neurodegeneration [4]. Microglial activation has been observed in idiopathic PD [5], but its role in the pathophysiology of genetic PD is unknown. A recent study using positron emission tomography (PET) imaging demonstrated increased microglial activation in the substantia nigra along with putaminal dopaminergic dysfunction in patients with idiopathic rapid eye movement sleep behavior disorder [6], a condition that may progress over time to a neurodegenerative alpha-synucleinopathy [7].

The present study aimed to investigate whether raised levels of microglial activation and alterations of the nigrostriatal dopaminergic function occur as well in LRRK2-NMCs.

## Methods

Eight LRRK2-NMCs were recruited from Hospital Clínic de Barcelona between September 2016 and May 2018. Inclusion of LRRK2-NMC was based on the absence of Parkinsonism and availability to perform the study. All NMC had a 60.5-min ^11^C-(R)-PK11195 PET scan (ECAT HRRT; CTI/Siemens, Knoxville, TN, USA) to assess levels of microglial activation (expressing 18-kDa translocator protein) and a 94.5-min ^18^F-DOPA PET scan to assess the integrity of the dopaminergic system. Each subject had a T1-weighted MRI (3 T MAGNETOM Skyra, Siemens Healthcare, Germany) performed for co-registration of PET images. PET findings were compared with those of 29 healthy controls (^11^C-PK11195 PET *n* = 20, ^18^F-DOPA PET *n* = 9). Control PET scans were acquired for two former projects using the exact same scanner and scan protocols [6, 8]. All participants were examined to exclude Parkinsonism. Assessments with the MDS-UPDRS part III, and Mini-Mental State Examination were also performed. All the assessments of this study were performed at the Department of Nuclear Medicine and PET Centre, Aarhus University Hospital.

Volumes of interest (VOI) sampled included the substantia nigra (^11^C-PK11195 PET only) plus putamen and caudate nucleus for both PET tracers. Image analysis was performed as previously described [6]. Briefly, parametric images of ^11^C-PK11195 binding potentials (BP_ND_) were generated using a simplified reference tissue model with an individual tissue non-specific input function extracted with a supervised cluster-analysis approach. Similarity between the ^11^C-PK11195 reference input function extracted from LRRK2-NMCs and controls was confirmed with a repeated measurement analysis (*χ*^2^ test, *p* > 0.05). In those VOI’s where ^11^C-PK11195 binding is lower than that of the selected reference tissue voxels, this model computes a negative BP_ND_. ^18^F-DOPA influx constant (Ki) images were generated using the Patlak graphical approach with the occipital lobe as the non-specific uptake reference region.

Group differences in ^11^C-PK11195 BP_nd_ and ^18^F-DOPA Ki were interrogated with unpaired Student’s *t* test (*p* < 0.05), normal distribution of data was checked with normal probability plots and D’Agostino–Pearson normality test. For descriptive purposes, individually *raised* regional ^11^C-PK11195 binding and *reduced* regional ^18^F-DOPA uptake was defined as a statistically significant deviation from the controls mean value (left and right side averaged) of two or more standard deviations (*z* score ≥ 2 and *z* score ≤ − 2)*.* Statistical analysis and graphical presentations were performed in Stata IC 14.2 (StataCorp LP, TX, USA) and GraphPad Prism 8 (GraphPad Software, La Jolla, CA, USA).

## Results

Eight LRRK2-NMCs, 7 men, with a mean UPDRS part III score of 2.6 (SD;1.6) and a mean age of 55.8 years (range 39.8–73.4), were compared to 20 ^11^C-PK11195 controls (12 men, mean age 66.8 years, range 58–80) and 9 ^18^F-DOPA controls (9 men, mean age 64.6 years, range 59.9–69.5). The left–right averaged putaminal ^18^F-DOPA uptake was significantly (*p* = 0.006) reduced by − 9.2% (range − 16.9% to 4.7%) in LRRK2-NMCs compared to controls, while caudate ^18^F-DOPA uptake was similar (*p* = 0.468). No group differences were observed in VOI analysis with ^11^C-PK11195 PET (substantia nigra *p* = 0.075, putamen *p* = 0.678, caudate nucleus *p* = 0.695).

**Fig. 1:**
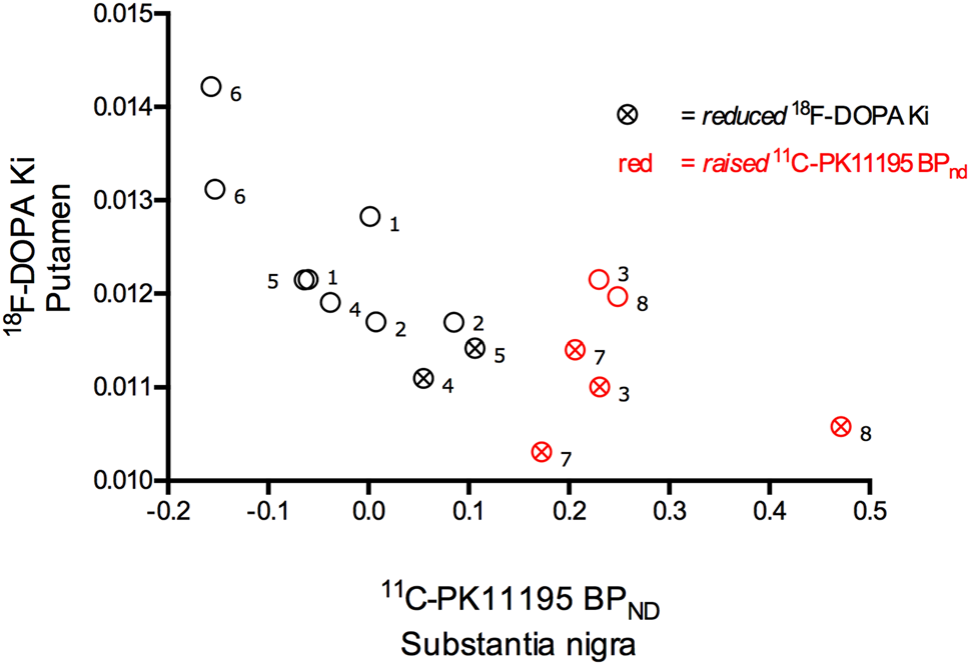
^18^F-DOPA uptake in putamen and ipsilateral ^11^C-PK11195 binding in substantia nigra in non-manifesting LRRK2 G2019S carriers. Values from left and right side in eight non-manifesting LRRK2 G2019S carriers are depicted. ⨂ indicates significantly reduced ^18^F-DOPA Ki values, which are two or more standard deviations below the average mean of controls (*z* score ≤ − 2). Red colour indicates significantly raised ^11^C-PK11195 BP_ND_ two or more standard deviations above the average mean of controls (*z* score ≥ 2). Observations from each individual carrier are marked with numbers from one to eight, which corresponds to their number in Table 1, individual analysis

Five out of eight LRRK2-NMCs had *reduced*^18^F-DOPA uptake in putamen (*z* score ≤ − 2), four unilateral and one bilateral (Table 1; Fig. 1). Three out of eight LRRK2-NMC had bilaterally *raised* levels of ^11^C-PK11195 binding in the substantia nigra (*z* score ≥ 2), coincidental with unilaterally *reduced* putaminal ^18^F-DOPA uptake (*z* score ≤ − 2) in two and bilaterally reduced in one (Table 1; Fig. 1). One subject had unilateral *raised* putaminal ^11^C-PK11195 binding without ipsilateral *reduced* putaminal ^18^F-DOPA uptake (Table 1).

**Table 1:** ^11^C-PK11195 BP_nd_ and ^18^F-DOPA Ki in controls and non-manifesting LRRK2 carriers

	^11^C-PK11195 BP_nd_		^18^F-DOPA Ki	
	Controls (*n* = 20)	LRRK2-NMCs (*n* = 8)	Controls (*n* = 9)	LRRK2-NMCs (*n* = 8)
Group analysis				
Substantia nigra	− 0.006 (0.09)	0.08 (0.16)	–	–
Putamen	0.08 (0.11)	0.06 (0.13)	0.0131 (0.00069)	0.0120* (0.00087)
Caudate	− 0.15 (0.12)	− 0.17 (0.13)	0.0113 (0.00085)	0.0120 (0.0017)

	*Reduced* putamen ^18^F-DOPA Ki	*Raised* substantia nigra ^11^C-PK11195 BP_nd_	*Raised* putamen ^11^C-PK11195 BP_nd_	UPDRS-III	MMSE
Individual analysis					
Carrier 1				0	28
Carrier 2				5	30
Carrier 3	○	●		4	30
Carrier 4	○			3	30
Carrier 5	○			2	29
Carrier 6				3	30
Carrier 7	●	●		3	29
Carrier 8	○	●	○	1	26

Values from both left and right side are averaged in the group analysis. Values are mean and standard deviation in brackets. *p < 0.05 in group analysis

Unilateral (○) or bilateral (●) *reduced* putaminal ^18^F-DOPA Ki value (*z* score ≤ − 2) and/or *raised* substantia nigra ^11^C-PK11195 BP_ND_ (*z* score ≥ 2) in individual carriers

*NMCs* non-manifesting carriers, *UPDRS-III* Unified Parkinson’s Disease Rating Scale—part three, *MMSE* Mini-Mental State Examination

## Discussion

This PET imaging study performed in LRRK2-NMCs found *reduced* putaminal ^18^F-DOPA uptake in five out of eight LRRK2-NMCs as previously reported in subjects without manifest PD carrying different subtypes of LRRK2 mutations [9]. Three carriers in our cohort had bilaterally raised substantia nigra ^11^C-PK11195 binding, indicating microglial activation, together with reductions in putaminal ^18^F-DOPA uptake (unilaterally *reduced* in two subjects and bilaterally in one). To our knowledge, this is the first report of activated microglia in LRRK2-NMC.

While not in all cases, a reduced putaminal ^18^F-DOPA uptake without increased ipsilateral nigral ^11^C-PK11195 binding was observed (subject 4 and 5; Fig. 1), suggesting that in LRRK2-NMC, dysfunction of putaminal dopaminergic axonal terminals may antedate microglial activation in nigral cell bodies, presumed to be related with neurodegeneration [4]. A similar pattern (normal range substantia nigra ^11^C-PK11195 binding and reduced ipsilateral putaminal ^18^F-DOPA uptake) has been reported previously in occasional subjects with prodromal PD [6] and manifest idiopathic PD patients [5]. The significance of microglial activation in the pathophysiology of PD is yet to be established. The findings in the current study, together with those found in a previous one examining prodromal PD [6] support the concept that similar alterations occur in the premotor phase in both genetic and idiopathic forms of the disease. Microglial cells are able to express either a neuroprotective or a cytotoxic phenotype [4]. Which of these is occurring in the examined LRRK2-NMC can only be determined with PET tracers binding specifically to each microglia phenotype; however, this kind of in vivo tracer is currently not available.

There are limitations to this study to be considered. First, the small number of cases examined may preclude the generalization of the results. Second, the LRRK2-NMC included in this study are younger than the controls which may account for a selection bias. Considering the age-related and low and penetrance of LRRK2 G20109S mutation [1, 2], it is possible that we have studied subjects who will never develop PD or at a very early phase of the disease when biochemical and pathological events have not yet started [10]. However, the alteration of ^18^F-DOPA PET in five LRRK2-NMC indicates that in most of LRRK-NMC in this cohort, pathological changes are already occurring. Given that age is the greatest risk factor for developing LRRK2-PD, we investigated whether PET findings were more likely to occur in the eldest LRRK2-NMC. We found that age did not correlate with PET alterations in the studied cohort, suggesting that other factors may influence ^18^F-DOPA dysfunction and microglial activation (individual data including age, not shown to avoid subject identification). Third, as previously reported in animal models [11], levels of microglial activation in PD may be influenced by the presence of pathological α-synuclein inclusions. In LRRK2 G2019S PD, a pleiotropic neuropathology has been reported, including subjects without Lewy-type α-synuclein pathology [3, 12]. This pathological variability may possibly influence the heterogeneity of the results. Finally, ^11^C-PK11195 may not only bind to the 18-kDa translocator protein located on mitochondria inside the activated microglial cells but also on astrocytes; however, observations show that this only accounts for minor degree of ^11^C-PK11195 signal [13].

This is the first study performed in LRRK2-NMCs showing dopamine dysfunction in five out of eight subjects and concomitant nigral microglial activation in three of them. These results suggest that PET imaging with ^18^F-DOPA and ^11^C-PK11195 could help identify NMCs with ongoing nigrostriatal pathology and show that at least in some cases, neuroinflammation could play a role in the pathophysiology of early phases of LRRK2-PD. The contribution of the observed in vivo microglial response and its clinical relevance at individual level still needs to be elucidated. Prospective studies using similar PET tracers in a larger study population with appropriate clinical follow-up may clarify these issues.

## References

[1] Healy DG, Falchi M, O'Sullivan SS, Bonifati V, Durr A, Bressman S, Brice A, Aasly J, Zabetian CP, Goldwurm S, Ferreira JJ, Tolosa E, Kay DM, Klein C, Williams DR, Marras C, Lang AE, Wszolek ZK, Berciano J, Schapira AH, Lynch T, Bhatia KP, Gasser T, Lees AJ, Wood NW, L.C. International (2008). LC International, Phenotype, genotype, and worldwide genetic penetrance of LRRK2-associated Parkinson's disease: a case–control study. Lancet Neurol.

[2] Marder K, Wang Y, Alcalay RN, Mejia-Santana H, Tang MX, Lee A, Raymond D, Mirelman A, Saunders-Pullman R, Clark L, Ozelius L, Orr-Urtreger A, Giladi N, Bressman S, L.A.J. Consortium (2015). Age-specific penetrance of LRRK2 G2019S in the Michael J. Fox Ashkenazi Jewish LRRK2 Consortium. Neurology.

[3] Kalia LV, Lang AE, Hazrati LN, Fujioka S, Wszolek ZK, Dickson DW, Ross OA, Van Deerlin VM, Trojanowski JQ, Hurtig HI, Alcalay RN, Marder KS, Clark LN, Gaig C, Tolosa E, Ruiz-Martinez J, Marti-Masso JF, Ferrer I, Lopez de Munain A, Goldman SM, Schule B, Langston JW, Aasly JO, Giordana MT, Bonifati V, Puschmann A, Canesi M, Pezzoli G, Maues De Paula A, Hasegawa K, Duyckaerts C, Brice A, Stoessl AJ, Marras C (2015). Clinical correlations with Lewy body pathology in LRRK2-related Parkinson disease. JAMA Neurol.

[4] Ransohoff RM (2016). How neuroinflammation contributes to neurodegeneration. Science.

[5] Gerhard A, Pavese N, Hotton G, Turkheimer F, Es M, Hammers A, Eggert K, Oertel W, Banati RB, Brooks DJ (2006). In vivo imaging of microglial activation with [^11^C](R)-PK11195 PET in idiopathic Parkinson's disease. Neurobiol Dis.

[6] Stokholm MG, Iranzo A, Ostergaard K, Serradell M, Otto M, Svendsen KB, Garrido A, Vilas D, Borghammer P, Santamaria J, Moller A, Gaig C, Brooks DJ, Tolosa E, Pavese N (2017). Assessment of neuroinflammation in patients with idiopathic rapid-eye-movement sleep behaviour disorder: a case–control study. Lancet Neurol.

[7] Iranzo A, Tolosa E, Gelpi E (2013). Neurodegenerative disease status and post-mortem pathology in idiopathic rapid-eye-movement sleep behaviour disorder: an observational cohort study. Lancet Neurol.

[8] Parbo P, Ismail R, Hansen KV, Amidi A, Marup FH, Gottrup H, Braendgaard H, Eriksson BO, Eskildsen SF, Lund TE, Tietze A, Edison P, Pavese N, Stokholm MG, Borghammer P, Hinz R, Aanerud J, Brooks DJ (2017). Brain inflammation accompanies amyloid in the majority of mild cognitive impairment cases due to Alzheimer's disease. Brain.

[9] Wile DJ, Agarwal PA, Schulzer M, Mak E, Dinelle K, Shahinfard E, Vafai N, Hasegawa K, Zhang J, McKenzie J, Neilson N, Strongosky A, Uitti RJ, Guttman M, Zabetian CP, Ding YS, Adam M, Aasly J, Wszolek ZK, Farrer M, Sossi V, Stoessl AJ (2017). Serotonin and dopamine transporter PET changes in the premotor phase of LRRK2 parkinsonism: cross-sectional studies. Lancet Neurol.

[10] Salat D, Noyce AJ, Schrag A, Tolosa E (2016). Challenges of modifying disease progression in prediagnostic Parkinson's disease. Lancet Neurol.

[11] Peelaerts W, Bousset L, Van der Perren A, Moskalyuk A, Pulizzi R, Giugliano M, Van den Haute C, Melki R, Baekelandt V (2015). alpha-Synuclein strains cause distinct synucleinopathies after local and systemic administration. Nature.

[12] Schneider SA, Alcalay RN (2017). Neuropathology of genetic synucleinopathies with parkinsonism: review of the literature. Mov Disord.

[13] Venneti S, Lopresti BJ, Wiley CA (2006). The peripheral benzodiazepine receptor (translocator protein 18 kDa) in microglia: from pathology to imaging. Prog Neurobiol.

